# Epigenetic regulation of TDP-43: potential implications for amyotrophic lateral sclerosis

**DOI:** 10.3389/fmmed.2025.1530719

**Published:** 2025-02-13

**Authors:** D. Y. Mengistu, M. Terribili, C. Pellacani, L. Ciapponi, M. Marzullo

**Affiliations:** ^1^ Department of Biology and Biotechnologies, Sapienza University of Rome, Rome, Italy; ^2^ Istituto Di Biologia e Patologia Molecolari, CNR, Sapienza Università Di Roma, Rome, Italy

**Keywords:** TDP-43, epigenetic regulation, ALS (amyotrophic lateral sclerosis), DNA methylation, histone modifications, microRNA

## Abstract

Amyotrophic lateral sclerosis (ALS) is a multifactorial neurodegenerative disease characterized by the progressive degeneration of motor neurons. One of the key pathogenic factors implicated in ALS is TDP-43 (TAR DNA-binding protein 43), an RNA-binding protein encoded by the *TARDBP* gene. Under normal physiological conditions, TDP-43 predominantly resides in the nucleus, where it plays a critical role in regulating gene expression, alternative splicing, RNA transport, and stability. In ALS, TDP-43 undergoes pathological mislocalization from the nucleus to the cytoplasm, disrupting its normal function and contributing to disease progression. The nuclear loss of TDP-43 leads to widespread dysregulation of RNA metabolism. Moreover, mislocalized TDP-43 aggregates in the cytoplasm, acquires toxic properties that sequester essential RNA molecules and proteins. Importantly, deviations in TDP-43 levels, whether excessive or reduced, can lead to cellular dysfunction, and contribute to disease progression, highlighting the delicate balance required for neuronal health. Emerging evidence suggests that epigenetic mechanisms may play a crucial role in regulating *TARDBP* expression and, consequently, TDP-43 cellular levels. Epigenetic modifications such as DNA methylation, histone modifications, and non-coding RNAs are increasingly recognized as modulators of gene expression and cellular function in neurodegenerative diseases, including ALS. Dysregulation of these processes could contribute to aberrant *TARDBP* expression, amplifying TDP-43-associated pathologies. This review explores and summarizes the recent findings on how specific epigenetic modifications influence TDP-43 expression and discusses their possible implications for disease progression.

## Overview of amyotrophic lateral sclerosis

Amyotrophic lateral sclerosis (ALS) is a progressive and still incurable neurodegenerative disease with an incidence of approximately 2 per 100,000 person-years (van Rheenen et al., 2021). ALS is characterized by the degeneration of both upper and lower motor neurons, denervation of voluntary muscles, progressive muscle atrophy, and a grim prognosis of 2–5 years from symptom onset to fatality (Hardiman et al., 2017). Although the disease can strike at any age, the majority of ALS patients are diagnosed between 50 and 75 years old (Wang et al., 2017). Approximately 5%–10% of ALS patients have a family history, with a monogenic mutation identified as the cause of familial ALS, while the remaining cases are sporadic. Currently, variants in more than 120 genes have been linked to ALS (Mathis et al., 2019; Chen et al., 2022; Nijs and Van Damme, 2024), including superoxide dismutase 1 (*SOD1*), chromosome 9 open reading frame 72 (*C9orf72*), Fused in Sarcoma (*FUS*), and TAR DNA binding protein (*TARDBP*, encoding TDP-43) (Al-Chalabi and Hardiman, 2013; Ling, 2018). TDP-43 is an RNA binding protein, known as a major pathogenic factor in ALS (El et al., 2015). It regulates gene expression by controlling RNA transcription, splicing, transport, and translation (Ma et al., 2021; Duan et al., 2022). Missense mutations in the *TARDBP* gene have been associated with ∼1%–5% of familial ALS (Sreedharan et al., 2008). These mutations lead to the formation of TDP-43 aggregates, mostly in the cytoplasm. Interestingly, it has been shown that in more than 95% of ALS cases, even without mutation in the *TARDBP* locus, the wild-type TDP-43 protein tends to form aggregates (Chen-Plotkin et al., 2010). Since TDP-43 accumulation is generally associated with its nuclear depletion, whether neurodegeneration results from the toxicity of cytoplasmic aggregates or the loss of TDP-43 nuclear function is still under debate (Lee et al., 2012; Xu, 2012; Robberecht and Philips, 2013; Cascella et al., 2016).

Epigenetic modifications begin to gain considerable attention in relation to neurodegenerative diseases due to their role in the regulation of neurophysiology associated genes (Bradley-Whitman and Lovell, 2013; Hou et al., 2019; Wilson et al., 2023).

Epigenetic modifications are reversible changes that affect gene expression without altering the DNA sequence (Waddington, 2012). The three primary epigenetic mechanisms are DNA methylation (DNAme), histone post-translational modifications (PTMs) and microRNAs (miRNAs) (Nakayama et al., 2001; Portela and Esteller, 2010; Yao et al., 2019).

While evidence remains limited, aberrant epigenetic pathways linked to ALS are beginning to be uncovered, suggesting that such mechanisms may directly or indirectly affect the expression of ALS-related genes, thereby potentially contributing to the disease phenotype (Berson et al., 2018).

Elevated global DNA methylation level has been identified in both spinal cord and blood samples of ALS patients compared to controls, indicating widespread epigenetic changes associated with the disease (Figueroa-Romero et al., 2012). Notably, demethylation in the promoters of key ALS-associated genes, including *SOD1*, *FUS*, *TARDBP*, and *C9orf72*, has been observed in both healthy and affected subjects (Coppedè et al., 2018), suggesting that major ALS causative genes undergo common epigenetic reprogramming.

In a blood-based epigenome-wide association study (EGWAS), involving 10,000 ALS patients and control subjects, 45 differentially methylated positions (DMPs) were identified as strongly associated with ALS (Hop et al., 2022). The analyses of overlapping DMP and loci enrichment, highlighted the involvement of metabolic, inflammatory, and cholesterol pathways in the ALS pathogenesis (Hop et al., 2022).

Data from a small cohort of ALS patients, revealed the enrichment of specific histone modifications (H3K9me3, H3K27me3, and H4K20me3) on *C9orf72* expanded repeats in brain tissue. These modifications, known to suppress gene expression, may contribute to the observed reduction in *C9orf72* mRNA levels in the frontal cortices and cerebella of ALS-affected patients (Belzil et al., 2013). Collectively, these data highlight the role of epigenetic mechanisms in regulating ALS-associated genes expression, whose alteration may be associated with the disease.

## Role of TDP-43 in ALS

TDP-43 is a highly conserved heterogeneous ribonucleoprotein (hnRNP) involved in mRNA transcription, translation, splicing, axonal transport, apoptosis, epigenetic modifications, and cryptic exon regulation (Jiang and Ngo, 2022). It is encoded by the *TARDBP* gene located on chromosome 1 and consists of 414-amino acids.

**FIGURE 1:**
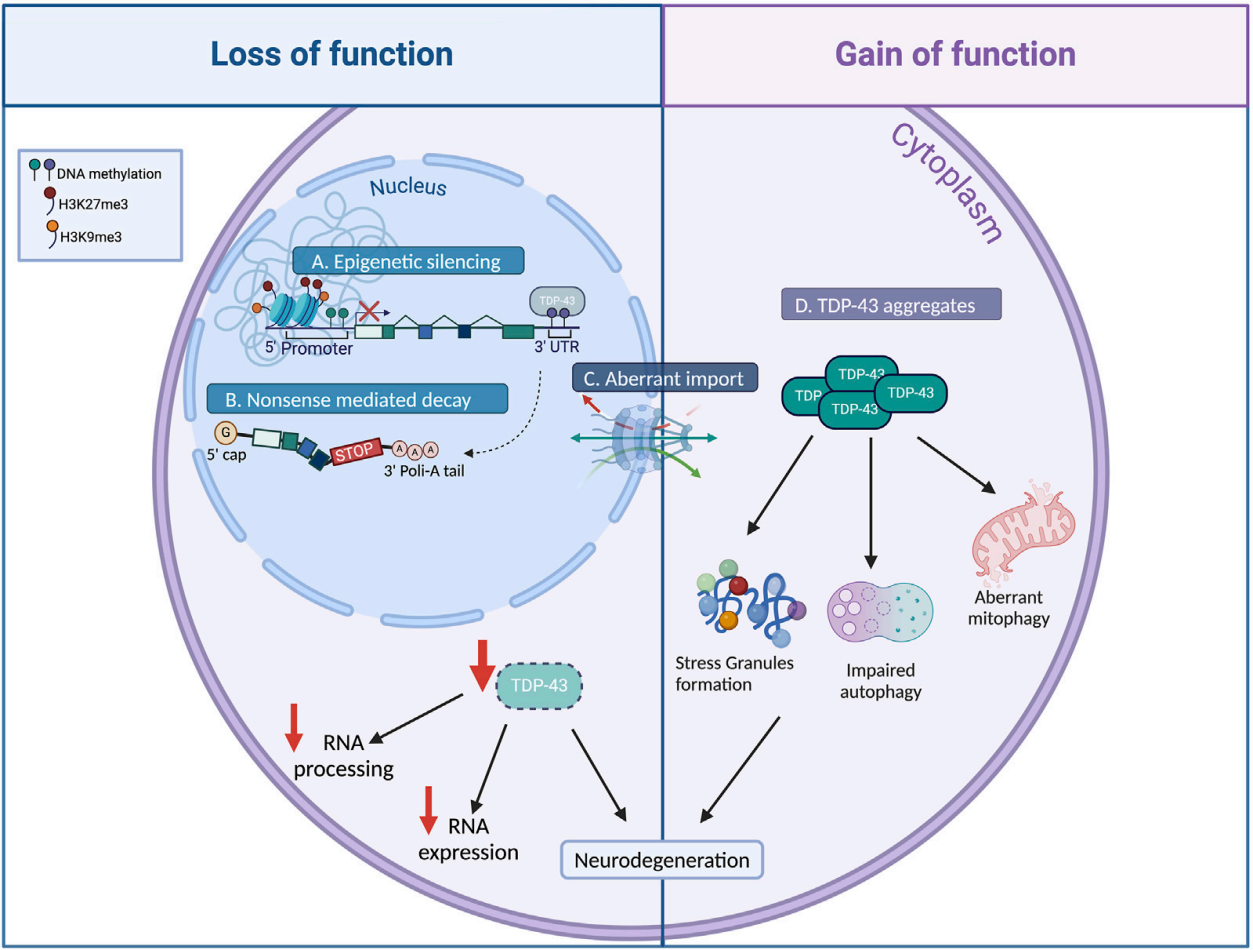
Causes and consequences of TDP-43 altered level. Various mechanisms have been identified to modulate TDP-43 cellular levels. Impairments in TDP-43 epigenetic regulation (A), alternative splicing autoregulation (B), and nucleocytoplasmic shuttling (C), result in TDP-43 nuclear depletion (loss of function), leading to dysregulation of RNA metabolism and gene expression, which contribute to neurodegeneration. Mutations in TDP-43 or abnormal post-translational modifications result in the formation of cytoplasmic TDP-43 aggregates (D, gain of function), inducing aberrant stress granules formation, mitochondrial dysfunction, and impaired autophagy ensuing neurodegeneration (Created with Biorender).

Both gain- and loss-of-TDP-43 function have been associated with ALS pathogenesis, however the exact contribution of each mechanism to neurodegeneration is still matter of debate (Figure 1; Lee et al., 2012). Mutations in TDP-43 seem to act mostly in a gain of function manner, affecting the basic biological properties of the protein and enhancing its tendency to form aggregates (Ratti and Buratti, 2016). The cytoplasmic aggregation of TDP-43 induce multiple cytotoxic effects, such as aberrant stress granule dynamics, liquid–liquid phase separation, mitochondrial dysfunction, endoplasmic reticulum (ER) stress, impaired axonal transport, and proteolysis dysfunction (Figure 1; Kabashi et al., 2010; McDonald et al., 2011; Barmada et al., 2014; Sun and Chakrabartty, 2017; Jiang and Ngo, 2022; Tamaki and Urushitani, 2022).

At the same time, the depletion of TDP-43 can result in deleterious consequences, including motor deficit, muscle degeneration and neuronal loss (Figure 1; Feiguin et al., 2009; Lu et al., 2009; Kabashi et al., 2010; Iguchi et al., 2013; Schmid et al., 2013; Vanden Broeck et al., 2013; Ratti and Buratti, 2016; Strah et al., 2020). Therefore, a combination of both gain- and loss-of-function mechanisms may be plausible in the onset and progression of ALS.

In line with the need to precisely regulate TDP-43 level within the cells, emerging evidence highlights the role of epigenetic mechanisms in controlling TDP-43 expression to maintain the correct protein amount during life and development of an individual. When these mechanisms become deregulated, such as during aging, it results in altered TDP-43 levels, leading to neurodegeneration (Pacetti et al., 2022; Marzullo et al., 2023).

This review explores recent findings on *TARDBP* epigenetic regulation, examining how DNA methylation, histone modifications, and microRNA precisely tune TDP-43 expression levels and how dysregulation of these processes may contribute to ALS pathogenesis.

## Epigenetic regulation of TDP-43

### DNA methylation patterns influence TDP-43 expression levels

DNA methylation is a crucial epigenetic mechanism that regulates gene expression by adding a methyl group to cytosine residues within CpG dinucleotides by DNMTs. Typically, hypermethylation at gene promoters represses gene expression, while methylation within coding regions can stimulate transcription and influence alternative splicing. DNA methylation affects transcription in two principal ways: directly, by impeding the binding of specific transcription factors to their target sequences (Hamidi et al., 2015) or indirectly, by recruiting histone deacetylases, leading to chromatin condensation (Rose and Klose, 2014; Hamidi et al., 2015; Yin et al., 2017; Baumann et al., 2019).

Recent studies highlight the role of DNA methylation in regulating *TARDBP* expression, influencing both its overall levels and the production of specific isoforms. Notably, age-related DNA demethylation at the *TARDBP* 3′UTR has been shown to reduce alternative splicing and increase *TARDBP* mRNA expression (Koike et al., 2021; Koike, 2024). However, there is conflicting evidence on whether TDP-43 expression levels increase or decrease during aging, leaving an open debate on this issue (Liu et al., 2015; Koike et al., 2021; Pacetti et al., 2022; Marzullo et al., 2023).

**FIGURE 2:**
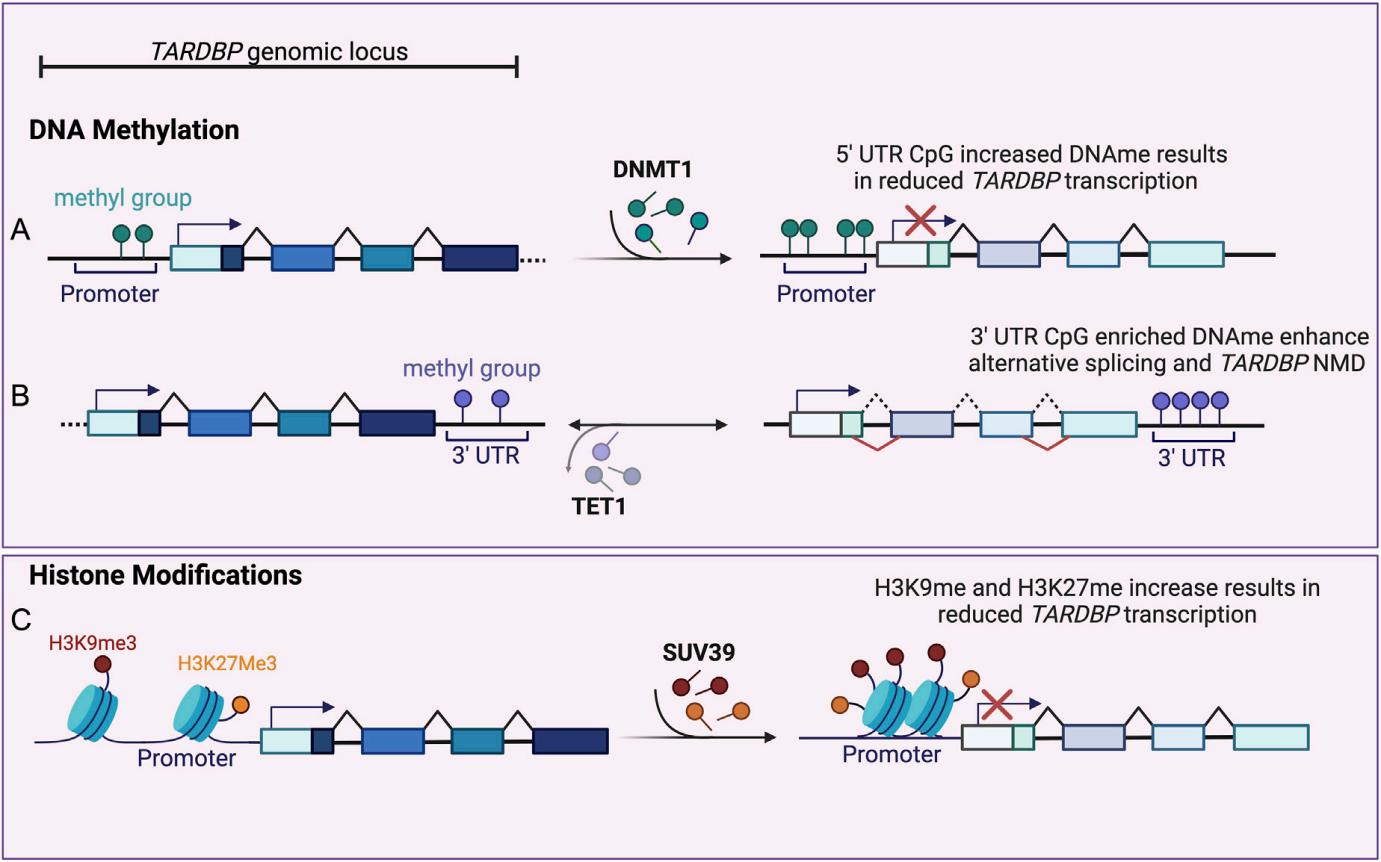
Epigenetic Regulation of *TARDBP*. **(A)** Increased CpG methylation (dark green circles) at *TARDBP* promoter, mediated by DNMT1 (right pointing arrow), inhibits *TARDBP* transcription (red cross), resulting in reduced TDP-43 levels (expressed as lighter exon colors). **(B)** Enrichment of methylated CpG sites (purple circles) within the *TARDBP* 3′ UTR genomic region, regulates alternative splicing (red lines) of introns 6 and 7. This leads to the inclusion of a premature stop codon and to *TARDBP* mRNA nonsense mediated decay (NMD), resulting in reduced TDP-43 levels (expressed as lighter exon colors). TET1 mediated 3′UTR CpG demethylation (left pointing arrow) reduces alternative splicing resulting in TDP-43 sustained expression (dark exon colors). **(C)** Increased H3K27 trimethylation (H3K27me3, yellow circles) and SUV39 mediated (right pointing arrow) H3K9 trimethylation (H3K9me3, red circles) at *TARDBP* promoter region induces chromatin repression and inhibition of *TARDBP* transcription (red cross), resulting in reduced TDP-43 level (expressed as lighter exon colors) (Created with Biorender).

Methylation profiling of the *TARDBP* gene revealed a cluster of five methylated CpG sites around the 5′splice site of intron 7 in the 3′UTR genomic autoregulatory region, which showed moderate to high methylation levels in the human prefrontal cortex (Koike et al., 2021). TDP-43 binds to its own pre-mRNA at this site and regulates the alternative splicing of introns 6 and 7, resulting in an isoform that is degraded via nonsense mediated decay (NMD; Figure 2B).

dCas9-Tet1 fusion protein has been used to effectively demethylate CpG sites in the *TARDBP* 3′UTR. Using this approach demethylation has been shown to suppress the alternative splicing of introns 6 and 7, leading to increased levels of unspliced, canonical *TARDBP* mRNAs. In contrast, targeting the *TARDBP* 3′UTR with dCas9-Dnmt3a did not affect DNA methylation or TDP-43 expression levels, suggesting that other enzymes may be involved in regulating methylation at these CpG sites (Koike et al., 2021).

Interestingly, strong age-dependent methylation decline at *TARDBP* 3′UTR CpGs has been shown in the motor cortex of healthy individuals, contributing to TDP-43 accumulation. This demethylation may increase the risk of ALS and explain the vulnerability of this tissue (Koike et al., 2021). However, the study has several limitations, including the small number of CpGs analyzed, the possibility of cell type mixing in the samples, and the need for larger, more diverse studies to validate the findings (Koike et al., 2021; Koike, 2024).

Conversely, studies in both *Drosophila* and mouse models have shown an age-dependent decrease of TDP-43 levels in the brain (Cragnaz et al., 2015; Marzullo et al., 2023; Huang et al., 2010; Liu et al., 2015). Pacetti and collegues showed that increased DNA methylation at specific sites within the *TARDBP* promoter region is associated with reduced TDP-43 expression in both mice and human cell lines, contributing to the tissue- and age-specific decline in TDP-43 protein levels. In mice, bisulfite sequencing revealed a correlation between increased methylation at four CpG-rich islands within the *TARDBP* 5′UTR and reduced TDP-43 levels in brain and skeletal muscle of 90-day-old mice (Figure 2A). However, no further significant TDP-43 reduction was observed in the brains of 360-day-old mice, suggesting that the decrease of TDP-43 expression in the brain stabilizes after a certain age (Pacetti et al., 2022). The same mechanism is also conserved in mouse motor neuron-derived NSC-34 cells, where a methylation pattern similar to that found in mouse brain and skeletal muscle was shown (Pacetti et al., 2022). Treatment of NSC-34 cells with the demethylating agent 5-azacytidine (5-AZA) reduced promoter methylation and significantly increased both TDP-43 mRNA and protein levels, suggesting that demethylation positively influences TDP-43 expression. Similar results were obtained when 5-AZA was applied to human SH-SY5Y cells, demonstrating the potential relevance of this mechanism for human TDP-43 regulation (Pacetti et al., 2022).

Knock down of DNA methyltransferases (DNMT1, DNMT3A, and DNMT3B) in NSC-34 cells also resulted in decreased promoter methylation and a corresponding significant increase in TDP-43 expression, further supporting the connection between reduced DNA methylation and elevated TDP-43 levels (Pacetti et al., 2022). In line with this, luciferase reporter assay showed that hypermethylation significantly suppressed *TARDBP* promoter activity, while the unmethylated promoter construct exhibited significantly higher activity, confirming that DNA methylation negatively regulates *TARDBP* gene expression (Pacetti et al., 2022).

Taken together, these findings strongly implicate DNA methylation as a crucial regulator of TDP-43 expression in both mice and humans, highlighting its potential contribution to the variations in TDP-43 levels observed during aging and in the context of ALS.

### Histone modifications control TDP-43 expression

Histone modifications are fundamental epigenetic regulators that plays a crucial role in modulating chromatin accessibility and gene expression (Cornett et al., 2016; Hyun et al., 2017). The precise role of histone modifications in regulating *TARDBP* expression is an evolving area of research. However, evidence indicates that these modifications, particularly at the *TARDBP* promoter, significantly influence TDP-43 levels, highlighting their potential implications for age-related neurodegeneration and ALS.

Increased levels of the repressive modification H3K27 trimethylation and decreased H2A.Z acetylation were observed at the *TARDBP* promoter in the brains of 90-day-old mice compared to 10-day-old controls (Figure 2C). These changes, along with increased DNA methylation and reduced RNA polymerase II occupancy, suggest a shift towards a more closed chromatin state in the *TARDBP* locus, which is associated with reduced TDP-43 expression levels.

In contrast to the brain, the liver exhibited reduced histone methylation and increased acetylated H2A.Z levels at both 10 and 90 days of age, suggesting a more open chromatin conformation at the *TARDBP* locus. Despite this open chromatin state, no significant changes in TDP-43 expression levels were observed in the liver. This discrepancy suggests that while chromatin accessibility might play a role in regulating *TARDBP* expression, other mechanisms, such as DNA methylation or TDP-43 autoregulation, may be more dominant in maintaining stable TDP-43 levels in the liver, highlighting the potential for tissue-specific regulatory mechanisms (Pacetti et al., 2022).

A recent study by Marzullo et al. (2023) demonstrated that in *Drosophila* brains, TBPH expression, the *Drosophila* ortholog of TDP-43, decreases with age. This age-related downregulation of TBPH is associated with reduced locomotor ability in the flies and is mediated by an epigenetic mechanism involving the histone methyltransferase Su(var)3–9 (Marzullo et al., 2023). Su(var)3-9 specifically mediates H3K9 trimethylation (H3K9me3) at the *TBPH* promoter, leading to reduced gene expression (Figure 2C). This mechanism of Su(var)3-9-mediated TDP-43 repression appears to be conserved across species, as evidence suggests similar activity in mice and human cells (Marzullo et al., 2023).

Su(var)3-9, also known as Suv39H1 in mammals, is a histone methyltransferase (HMT) responsible for the transfer of a methyl group to lysine 9 of histone H3. While traditionally associated with constitutive heterochromatin, emerging evidence points to a broader role for Su(var)3-9 in regulating gene expression to control cell differentiation and cell fate (Snigdha et al., 2016; Padeken et al., 2022). Modulation of Su(var)3-9 levels correlates with changes in TDP-43 expression. It was shown that increase of Su(var)3-9 activity, specifically in neurons, is sufficient to induce a reduction in TDP-43 expression and early locomotion defects in flies. Conversely, Su(var)3-9 loss prevents age-dependent H3K9me3 deposition at the *TBPH* locus, and results in increased TDP-43 expression, and consequently improved motility in old flies (Marzullo et al., 2023). Notably, the impact of Su(var)3-9 on TDP-43 expression and locomotion appears to be specific, as the loss of the other two histone methyltransferases, G9a and Eggless, does not affect either TDP-43 levels or locomotion (Marzullo et al., 2023).

Analogously to the *Drosophila* data, SUV39H1 depletion in human cells (HaCaT) correlates with reduced H3K9me3 at *TARDBP* promoter and corresponds to an increase in TDP-43 protein expression. Moreover, aging mimicking in human cells by H_2_O_2_ treatment, causes a significant reduction in TDP-43 expression which is prevented by the CRISPR knock out of the SUV39H1 gene, indicating that TDP-43 age-dependent epigenetic regulatory mechanisms is conserved in human cells (Marzullo et al., 2023).

These data underline the role of the epigenetic enzyme Su(var)3-9 in regulating the expression of the key ALS-related gene *TARDBP*, highlighting the presence of a conserved epigenetic mechanism that could potentially influence ALS pathogenesis.

### Role of non-coding RNAs in TDP-43 regulation

MicroRNAs (miRNAs) have emerged as important regulators of *TARDBP* expression, particularly through their interactions with the 3′UTR of *TARDBP* mRNA, influencing TDP-43 levels and potentially contributing to the pathogenesis of ALS.

miRNAs are small non-coding RNAs that regulate gene expression by binding to target messenger RNAs (mRNAs), leading to their degradation or inhibiting their translation (Morlando et al., 2013).

Deregulation of numerous miRNAs has been associated with ALS pathogenesis (Juźwik et al., 2019; Strong et al., 2024). TDP-43 and FUS, two of the major ALS causing genes, encode for ribonucleoproteins involved in miRNA biogenesis. TDP-43 regulates miRNA processing by interacting with DROSHA and DICER complexes and with several pri- and pre-miRNAs (Kawahara and Mieda-Sato, 2012), while FUS facilitates miRNA processing by co-transcriptionally recruiting DROSHA on the chromatin and binding to specific nascent pri-miRNA (Morlando et al., 2012). These functions have been linked to the vast downregulation of miRNAs observed in the spinal cord and motor neuron of ALS affected patients (Bicker and Schratt, 2015; Emde et al., 2015; Campos-Melo et al., 2018).

Few studies have looked at how miRNAs regulate TDP-43 expression. It was found that two miRNAs, miR-194 and miR-b2122, are significantly reduced in patients with sporadic ALS and that this reduction lead to higher levels of ALS-related mRNAs, including *TARDBP*, *FUS,* and *RGNEF* (Hawley et al., 2017).

A screening for miRNA involved in ALS pathogenesis identified four miRNAs (miR-27b-3p, miR-30a-5p, miR-181c-5p and miR-425–3p) that are downregulated after TDP-43 knockdown. Interestingly, miR-27b-3p and miR-181c-5p bind two conserved MREs (microRNA recognition elements) in the 3′UTR of *TARDBP* mRNA. Both these miRs are expressed in human spinal motor neurons and are significantly downregulated in the spinal cord of ALS patients (Hawley et al., 2020). miR-27b-3p and miR-181c-5p induce reduction of TDP-43 expression by interacting with the *TARDBP* 3′UTR mRNA and mediating its degradation. Interestingly, these two miRNAs are involved in a nuclear localization-dependent negative feedback loop with TDP-43. Cellular stress, which reduces the nuclear localization of TDP-43 leads to downregulation of miR-27b-3p and miR-181c-5p, further enhancing its accumulation by suppressing the miR mediated degradation. Specifically, TDP-43 knockdown reduces cytoplasmic mature levels of pre-miR-181c, suggesting a role of TDP-43 in DROSHA-mediated processing of pre-miR-181c-5p. In contrast, knockdown of TDP-43 causes only a reduction in mature miR-27b-3p without affecting pri- and pre-miRNAs, indicating that TDP-43 probably promotes DICER processing of pre-miR-27 b (Hawley et al., 2020). These results suggest that a reduction in nuclear levels of TDP-43 due to cellular stress leads to reduced miRNA levels, resulting in derepression of *TARDBP* transcription and consequent increased levels of TDP-43.

This biological mechanism may have implications for the pathogenesis of ALS, where reduced miRNA levels correspond to the upregulation and delocalization of TDP-43.

Of note, these studies on miRNA-mediated regulation of *TARDBP* expression were conducted in the HEK293T cell line, which may not fully recapitulate the molecular pathways found in motor neurons. Further studies using models of motor neuron dysfunction, both *in vitro* and *in vivo*, are needed to fully understand the role of this pathway in ALS pathogenesis.

## Conclusion

The epigenetic regulation of ALS associated genes is an emerging field, and our understanding remains limited. The question of whether ALS pathogenesis is driven by TDP-43 gain-of-function, loss-of-function, or a combination of both, remains an open question, highlighting the importance of studying mechanism involved in *TARDBP* expression including epigenetic regulation. Recent studies have begun to uncover the epigenetic mechanisms controlling *TARDBP* expression, and consequently TDP-43 levels (Table1), but there are still many unresolved discrepancies and no definitive evidence of a conserved mechanism across tissues and species.

**TABLE 1:** Epigenetic modifications affecting TDP-43 expression.

Epigenetic modification	*TARDBP* targeted site	Effect on TDP-43 expression	References
DNA methylation	5′UTR and promoter	Inhibition of *TARDBP* transcription ↓TDP-43	Pacetti et al. (2022)
	3′UTR	*TARDBP* mRNA Nonsense mediated decay ↓TDP-43	Koike et al. (2021)
H3K9me3	promoter	Inhibition of *TARDBP* transcription ↓TDP-43	Marzullo et al. (2023)
H3K27me3	5′UTR and promoter	Inhibition of *TARDBP* transcription ↓TDP-43	Pacetti et al. (2022)
acH2A.z	5′UTR and promoter	Enhance recruiting of RNAPII level on *TARDBP* promoter region ↑TDP-43	Pacetti et al. (2022)
mir-27b-3p and mir-181c-5p	mRNA 3′UTR	Mediate *TARDBP* mRNA silencing ↓TDP-43	Hawley et al. (2020)

In particular several studies focused on how *TARDBP* epigenetic regulation changes with age affecting the expression of TDP-43. In mouse brains, a decline in TDP-43 expression, is accompanied by increased DNA and H3K27 methylation on the *TARBDP* promoter in 90-day-old mouse brains, but no further decrease in TDP-43 levels in 360-day-old mouse brains (12-month-old) (Pacetti et al., 2022). In contrast, studies in *Drosophila* report a consistent reduction in TDP-43 levels throughout the fly lifespan, driven by aging-dependent H3K9me3 deposition at the *TARDBP* promoter, catalyzed by Su(var)3-9. This role of Su(var)3-9 seems to be evolutionarily conserved from *Drosophila* to vertebrates, as human cells depleted for Suv39H1 show loss of repressive H3K9me3 on *TARDBP* promoter and increased TDP-43 expression. The fly data indicate a direct correlation between epigenetic regulation of *TARDBP*, TDP-43 levels and aging-dependent locomotor decline (Cragnaz et al., 2015; Marzullo et al., 2023).

The divergence between the evidence from *Drosophila* and mice regarding TDP-43 decrease during aging and locomotion decline, could be explained by the differences in complexity and aging processes of the two organisms. It is essential to consider that 12-month-old mice (360 day-old) are commonly categorized as middle-aged, with mice between 18 and 22 months generally considered old (Yanai and Endo, 2021). Accordingly, the decline in mice movement capability initiates from 12 months onward, and more pronounced phenotypes are observed at 20 months of age, suggesting that relatively narrow age differences can produce significant behavioral differences during adulthood in mice (Mitchell et al., 2015; Sukoff Rizzo and Crawley, 2017). In *Drosophila* the authors were able to monitor the decrease of TDP-43 levels all over the fly lifespan and to drive a direct correlation between TDP-43 levels and aging-dependent locomotor decline. It is plausible that the 2 mouse aging points tested by Pacetti and collaborators, (90 days and 360 days) correspond to early and middle-age time points, were the increased DNA and H3K27me3 on the *TARBDP* promoter may not be sufficient to induce a further TDP-43 reduction and cause locomotion defects. To reconcile this discrepancy with the *Drosophila* data, it would be necessary to analyze *TARBDP* epigenetic profile and expression levels at later stages, specifically in 18/24-month-old mice, which is the widely recognized period for aging in mice.

Recent studies also showed that in the human motor cortex, *TARDBP* 3′UTR undergoes demethylation with aging, leading to increased expression of canonical *TARDBP* mRNA. Even though these observations seem in contrast with the aging-associated TDP-43 reduction described before, they highlight again a direct correlation between DNA methylation status, TDP-43 expression, and ALS-associated phenotypes (Koike et al., 2021). Given the complexity of TDP-43 regulation, it is conceivable to hypothesize the presence of different epigenetic mechanisms dictating its expression and stability, including microRNAs-mediated control.

The variability in TDP-43 levels influenced by diverse epigenetic modifications may contribute to differences in compensatory abilities for TDP-43 loss due to aggregate sequestration. Comprehending the regulation of the *TARDBP* locus and identifying all possible players affecting its epigenetic status and subsequent expression levels are key aspects in understanding the mechanisms underlying ALS pathogenesis or predisposition to aging-associated neurodegenerative diseases.

Individuals with reduced TDP-43 levels in the brain alongside pathological aggregates may be predisposed to ALS. The characterization of TDP-43 epigenetic control and the modulation of TDP-43 levels represent a key step toward the identification of a new potential mechanism to restore TDP-43 functionality in ALS pathogenesis.

Alteration in the epigenetic profile of *TARDBP* locus could represent an early stage of de-regulation of this ALS-related gene, thus representing a new promising target of therapy.

The reversible nature of epigenetic modifications and their accessibility to manipulation make them attractive targets for developing prognostic and therapeutic tools for ALS. Targeting these modifications could potentially restore proper *TARDBP* expression levels and ameliorate disease progression. However, extensive studies are crucial to identify a unifying epigenetic mechanism controlling *TARDBP* expression across diverse ALS subtypes and genetic backgrounds.
